# Comparative miRNA transcriptomics of macaques and mice reveals *MYOC* is an inhibitor for *Cryptococcus neoformans* invasion into the brain

**DOI:** 10.1080/22221751.2022.2081619

**Published:** 2022-06-04

**Authors:** Hailong Li, Xiaoxu Han, Wei Du, Yang Meng, Yanjian Li, Tianshu Sun, Qiaojing Liang, Chao Li, Chenhao Suo, Xindi Gao, Yu Qiu, Wen Tian, Minghui An, Hui Zhang, Yajing Fu, Xiaolin Li, Tian Lan, Sheng Yang, Zining Zhang, Wenqing Geng, Chen Ding, Hong Shang

**Affiliations:** a NHC Key Laboratory of AIDS Immunology, National Clinical Research Center for Laboratory Medicine, The First Affiliated Hospital of China Medical University, Shenyang, People’s Republic of China; b College of Life and Health Sciences, Northeastern University, Shenyang, People’s Republic of China; c Medical Research Centre, State Key Laboratory of Complex Severe and Rare Diseases, Peking Union Medical College Hospital, Chinese Academy of Medical Science, Beijing, People’s Republic of China; d Beijing Key Laboratory for Mechanisms Research and Precision Diagnosis of Invasive Fungal Diseases, Beijing, People’s Republic of China

**Keywords:** Cryptococcal meningoencephalitis, HIV/AIDS, brain dissemination, host-pathogen interactions, macaque, miRNA transcriptome, cytoskeleton, MYOC

## Abstract

Cryptococcal meningoencephalitis (CM) is emerging as an infection in HIV/AIDS patients shifted from primarily ART­naive to ART-experienced individuals, as well as patients with COVID-19 and immunocompetent hosts. This fungal infection is mainly caused by the opportunistic human pathogen *Cryptococcus neoformans*. Brain or central nervous system (CNS) dissemination is the deadliest process for this disease; however, mechanisms underlying this process have yet to be elucidated. Moreover, illustrations of clinically relevant responses in cryptococcosis are currently limited due to the low availability of clinical samples. In this study, to explore the clinically relevant responses during *C. neoformans* infection, macaque and mouse infection models were employed and miRNA-mRNA transcriptomes were performed and combined, which revealed cytoskeleton, a major feature of HIV/AIDS patients, was a centric pathway regulated in both infection models. Notably, assays of clinical immune cells confirmed an enhanced macrophage “Trojan Horse” in patients with HIV/AIDS, which could be shut down by cytoskeleton inhibitors. Furthermore, myocilin, encoded by *MYOC*, was found to be a novel enhancer for the macrophage “Trojan Horse,” and an enhanced fungal burden was achieved in the brains of *MYOC*-transgenic mice. Taken together, the findings from this study reveal fundamental roles of the cytoskeleton and *MYOC* in fungal CNS dissemination, which not only helps to understand the high prevalence of CM in HIV/AIDS but also facilitates the development of novel therapeutics for meningoencephalitis caused by *C. neoformans* and other pathogenic microorganisms.

## Introduction

Fungal invasive diseases are an increasing threat to global public health and lead to more than one million deaths annually, mainly caused by species of *Candida albicans*, *Aspergillus fumigates* and *Cryptococcus neoformans*. Cryptococcal meningoencephalitis (CM) is the leading disease among fungal meningoencephalitis and causes approximately 181,000 deaths each year, with mortality rates of 100% if untreated [1]. Recent studies showed that the prevalence and mortality of CM have not decreased and consequently appeals for action on this disease have been raised [2,3]. Additionally, CM is a major risk for patients with HIV/AIDS, with a prevalence of 77%–90% and leading to approximately 15% of deaths of patients with HIV/AIDS annually [4]. In the era of ART (antiretroviral therapy), CM is also an emerging infectious disease, shifting from primarily ART­naive individuals to more than 50% of cases being in ART-treated patients [2]. Seriously, the morbidity of cryptococcosis in immunocompetent individuals is increasing rapidly in China, Australia, Canada, and other countries and regions [4–8]. Importantly, some patients with COVID-19 suffered from secondary *Cryptococcus* infections, suggesting cryptococcosis is an important issue in the post-COVID-19 era [9–12]. Fungal CNS dissemination, which is vital for both fungal colonization and fungal clearance, is the lethal part of the infection process, but the mechanisms of CNS dissemination are lacking from *in vivo* or clinical studies.

To date, massive research has focused on CNS invasion during fungal meningitis and has achieved numerous milestones, including that cryptococci interact with brain epithelial cells directly and indirectly by microscopy or electron microscopy [13–16]. These studies were mainly performed on cell lines, mice, zebra fish or rabbits [17]; however, clinically relevant evidence is limited due to the unavailability of clinical samples. Cryptococcal meningoencephalitis is predominantly prevalent among immunocompromised individuals, especially those with HIV/AIDS [18–20]. Thus, elucidating mechanisms from clinical patients with HIV/AIDS is the most direct approach to understand *Cryptococcus* meningoencephalitis. A dysfunctional cytoskeleton of immune cells is a major pathological feature in patients with HIV/AIDS [21]. Meanwhile, previous studies indicated that *C. neoformans* infections also disturbed cytoskeleton pathways in mice and cell lines [22,23]. However, there is currently no evidence demonstrating mechanisms between the dampened cytoskeleton and cryptococci brain dissemination. Identification of relationships between the cytoskeleton and fungal infections may reveal novel mechanisms of fungal pathogenesis and provide targets for drug development in mycosis.

Host responses during infectious diseases are often divided into DNA, mRNA and protein levels, and also post-transcriptional and post-translational modifications [22,24,25]. Previous studies have identified many responses at mRNA, protein and post-translational levels, including key pathways and modulators, such as mineral metabolism, IL-17 signalling pathway, sugar metabolisms, OCSTAMP, IL-5, IL-13 and IL-17A [22,24,26–31]. Post-transcriptional modifications, assumed by miRNAs, also play important roles in mRNA and protein biosynthesis. Recently, several studies have demonstrated functions of non-coding RNAs during *C. neoformans* infections *in vitro*, but there is limited information *in vivo* [32–35].

In this study, to mimic human responses, macaques were employed and an *in vivo* miRNA-mRNA network was constructed. The cytoskeleton pathway was revealed as the core pathway regulated by *C. neoformans* in both macaques and mice. Moreover, clinical immune cell assays confirmed an enhanced macrophage “Trojan Horse” in patients with HIV/AIDS, and intervention of the cytoskeleton pathway disrupted the “Trojan Horse.” Furthermore, the cytoskeleton-associated gene, *MYOC*, was identified as an important factor for “Trojan Horse” by THP-1 cells and transgenic mice. Collectively, these findings demonstrate global responses at the miRNA-mRNA regulatory level, reveal novel modulators for fungal invasion, and may facilitate the development of novel therapeutics for fungal infectious diseases.

## Materials and methods

### Ethics statement

All work with human cells was reviewed and approved by the Medical Research Ethics Committee of the First Affiliated Hospital of China Medical University (2021-63-2). Animal infection experiments in macaques and mice were reviewed and ethically approved by the Research Ethics Committees of the College of Life and Health Sciences of Northeastern University (16099M) and Wincon TheraCells Biotechnologies Co., Ltd. (WD-20,150,701-a). All animal experiments were conducted according to the Guide for the Care and Use of Laboratory Animals issued by the Ministry of Science and Technology of the People’s Republic of China.

### Animal infection

Macaques and mice were purchased from Grandforest Co. (Guangxi, China) and Changsheng Biotech (China), respectively, and infections were performed as previously described [22]. Briefly, six female macaques were divided into two groups. Macaques were anesthetized by intraperitoneal injection (IP) with ketamine (10 mg·kg^−1^) and then infected via intratracheal injection with 10^8^ cells·mL^−1^ *C. neoformans* H99. Controls were injected with the same volume of PBS. Mice were anesthetized and infected intranasally with 10^5^ fungal cells. Macaques and mice were monitored for signs of infection and humanely killed at day 7 or 14 post-infection, or used for survival rate determination.

### miRNA sequencing and analysis

Total RNA of lung tissues was isolated using TRIzol and assessments of RNA were performed using a NanoDrop 8000. Small RNA-Seq libraries were prepared by using TruSeq® Small RNA Library Prep Kit according to the manufacturer’s protocol. Construction and sequencing of miRNA libraries were entrusted to Shanghai Personal Biotechnology Co., Ltd. (China), and then single-end sequencing was conducted using an Illumina NextSeq 500 platform. Raw data were obtained, and clean and unique reads were mapped to corresponding genomes by Bowtie. The expression of miRNAs was identified by using quantifier.pl in Mirdeep2 based on miRBase21. Differentially expressed miRNAs were calculated by DESeq.

### Quantitative PCR of miRNAs and mRNAs

For miRNAs, total miRNAs were isolated by miRNeasy Mini Kit (QIAGEN) according to the manufacturer’s instructions. First-strand cDNA was synthesized using random oligonucleotides and a miRcuRY LNA Universal RT microRNA PCR Universal cDNA Synthesis Kit II (EXIQON, USA) and U6 was employed as an internal reference. Primers (Table S5) were designed by using miRprimer and the optimum primer pairs were selected [36]. RT-qPCR was performed by using miRcuRY LNA Universal RT microRNA PCR Exilent SYBR master mix (EXIQON) and a StepOne Plus system. For mRNAs, total RNAs were isolated and cDNAs were synthesized. RT-qPCR was performed by using GoTaq®qPCR Master Mix (Promega) and the Roche LightCycler® 480 system.

### Histopathology, colony forming units (CFU) and survival rates assessments

Lung tissues from macaques and mice were collected as described previously [22]. For histopathology analyses, tissue samples were fixed with paraformaldehyde, frozen, and processed using a cryostat microtome (CM1850, Leica). Tissue sections of 10 µm thickness were stained with mucicarmine or haematoxylin/eosin. For CFU determination, homogenized lung and brain tissues were diluted, spread on YPD plates and incubated at 30 °C for two days, then colonies were counted and calculated. For determination of survival rates, C57BL/6 mice were divided randomly. Bodyweight was examined post infections. A decrease of 15% of initial body weight was identified as the physiological endpoint.

### Construction of MYOC-overexpressing THP-1 cell line and differentiation of THP-1-derived macrophages (TDMs)

The *MYOC* gene was cloned into the pCDH-EF1*α*-MCS-T2A-Puro lentiviral expression plasmid. Lentivirus was packaged by using jetPRIME® DNA & siRNA Transfection Reagent (polyplus) according to manufacturer’s instructions. Lentiviral infections of THP-1 cells were performed by centrifugation at 1200*g* at 37 °C in 24-well plates for 2 h. Screening of cells for puromycin resistance was conducted 72 h post-transduction for 2 weeks with 5 µg·mL^−1^ puromycin. TDMs were differentiated for 48 h in RPMI-1640 medium (10% foetal bovine serum (FBS)) containing 1% penicillin/streptomycin and 250 ng·mL^−1^ PMA (Phorbol 12-myristate 13-acetate, PMA) (Sangon Biotech, China).

### Isolation of human monocytes and induction of monocyte-derived macrophages (MDMs)

Peripheral blood mononuclear cells (PBMCs) from patients with HIV and healthy individuals were isolated by Ficoll-Paque PLUS density gradient media (Cytiva). Blood samples were centrifuged in a swing bucket rotor at 400*g* for 30 min at 25 °C with acceleration set at 5 and break at 0, followed by purification in cold PBS twice and centrifugation at 300*g* for 10 min at 4 °C with acceleration and break set at 5. The isolated PBMC were seeded into 48-well plates at 1 × 10^6^ cells per well in RPMI-1640 media without FBS for 1 h to allow adherence of monocytes, then the medium was exchanged for fresh RPMI-1640 supplemented with 10% FBS, 1% penicillin/streptomycin and 50 ng·mL^−1^ M-CSF. The medium was further renewed on days 3 and 6 during differentiation of the monocytes, and the MDMs were ready to use on day 7.

### Western blotting and antibodies

Western blotting of MYOC was performed using the standard protocol and antibodies used in this study were as follows: Anti-MYOC rabbit polyclonal antibody, Sangon Biotech, Order No. D227350; Mouse anti human GAPDH Loading Control Monoclonal Antibody (GA1R), DyLight™ 680, Invitrogen, Product No. MA5-15,738-D680; and Goat anti-Rabbit IgG H&L (HRP) Secondary Antibody, abcam, Cat No. ab6721.

### Phagocytosis effectivity, killing and transmigration assessments

MDMs or TDMs were seeded on 48-well plates at 1 × 10^5^ cells per well 24 h before fungi interaction. GFP-expressing strains of *C. neoformans* H99 were incubated overnight at 30 °C, washed, and opsonized by 18B7 (1mg·L^−1^) at room temperature for 30 min. Fungal cells and macrophages were incubated overnight in a CO_2_ incubator at a multiplicity of infection (MOI) of 1:10. The cells were then washed five times with PBS, digested with trypsin, and phagocytosis effectivity was detected by flow cytometry. For killing assays, the supernatant was collected after incubation and cells were washed and collected. Total lysates and supernatants were mixed, diluted, spread on YPD agar plates and incubated for 48 h at 30 °C, then colonies were counted and calculated. Migration of THP-1 cells and MDMs were performed by trans-well assay.

### Microscopy

Phagocytes of TDMs contained with *C. neoformans* were washed, and then fixed using ice-cold 4% paraformaldehyde (PFA) in 0.1 M PBS for 30 min room temperature, and stained for F-actin (phalliodin-Alexa647, A22287, Invitrogen) for 30 min at room temperature. Imaging was conducted using the laser confocal microscope ZEISS LSM 980 with Airyscan 2.

### Construction of MYOC-transgenic mice

MYOC-transgenic mice were produced by Beijing View Solid Biotechnology, China. The plasmid pCAG-MYOC was linearized by *BstEII* restriction enzyme (NEB) digestion, purified, and injected into zygotes of C57BL/6 mice in M2 media (Millipore) using a FemtoJet micromanipulator (Eppendorf, Germany). Microinjected zygotes were transferred into pseudo-pregnant female mice. All mice were maintained in a specific pathogen-free facility. Genotype identification was performed by PCR and sequencing from 2-week-old newborn mice (Table S5). Transgenic mice were mated with wild-type C57BL/6 mice to obtain heterozygous mice and colony expansion.

### Bioinformatics analysis

Targeted genes of miRNAs were predicted using miRWalk 3.0 [37]. Regulatory network miRNA-mRNA was constructed by Cytoscape. Gene ontology and Kyoto Encyclopedia of Genes and Genomes (KEGG) analyses were performed using R version 4.1.2, clusterProfiler v4.2.0, *org.Hs.eg.db* version 3.14.0 and *org.Mm.eg.db* version 3.14.0 packages and were plotted by ggplot2 version 3.3.5. KEGG of miRNAs was performed by using the web-based application miEAA [38] and were plotted by ggplot2. A heatmap of expression was generated by Pheatmap version 1.0.12. Homology analysis was performed based on the miRbase search engine database (https://www.mirbase.org/search.shtml).

### Statistics and reproducibility

All experiments were performed at least biological triplicates to ensure reproducibility. Statistics of phagocytosis effectivity, RT-qPCR and CFU determinations were calculated with an unpaired or paired student *t*-test using GraphPad Prism 9.0. All results were shown as mean ± standard error of the mean (SEM). When the *p*-value was less than 0.05, statistical significance was recognized.

## Results

### MicroRNA transcriptomics in macaque and mouse during cryptococcal pneumonia

**Figure 1.**
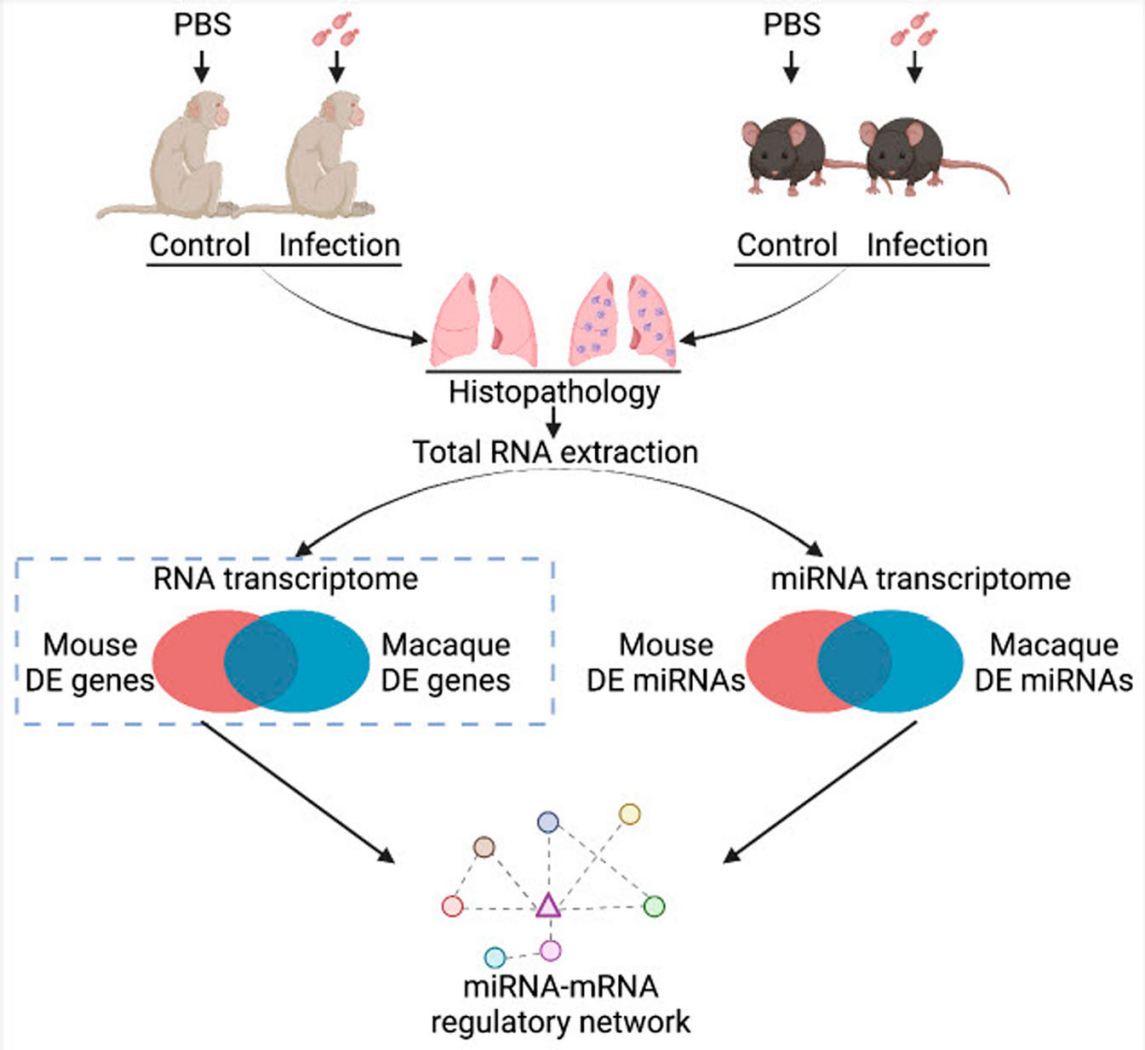
Flow chart for animal infections and RNA sequencing. Six macaques and mice were randomly divided into two groups and infected by *C. neoformans* H99 intranasally. Lung tissues were isolated 7 days post-infection. Total RNAs were isolated for mRNA-Seq and miRNA-Seq followed by confirmation of histopathology. A differentially expressed miRNA-mRNA regulatory network was subsequently constructed. This figure was created with BioRender.com.

Transcriptional responses during *C. neoformans* infections in macaque and mouse were unveiled in a previous study by our group and several regulators and pathways were identified [22]. To investigate global responses at post-transcriptional level, miRNA transcriptomes were performed in the current study using lung tissues isolated from macaque and mouse infection models (Figure 1). Furthermore, a miRNA-mRNA network was constructed to reveal a comprehensive and clinically relevant responses in cryptococcosis.

**Figure 2.**
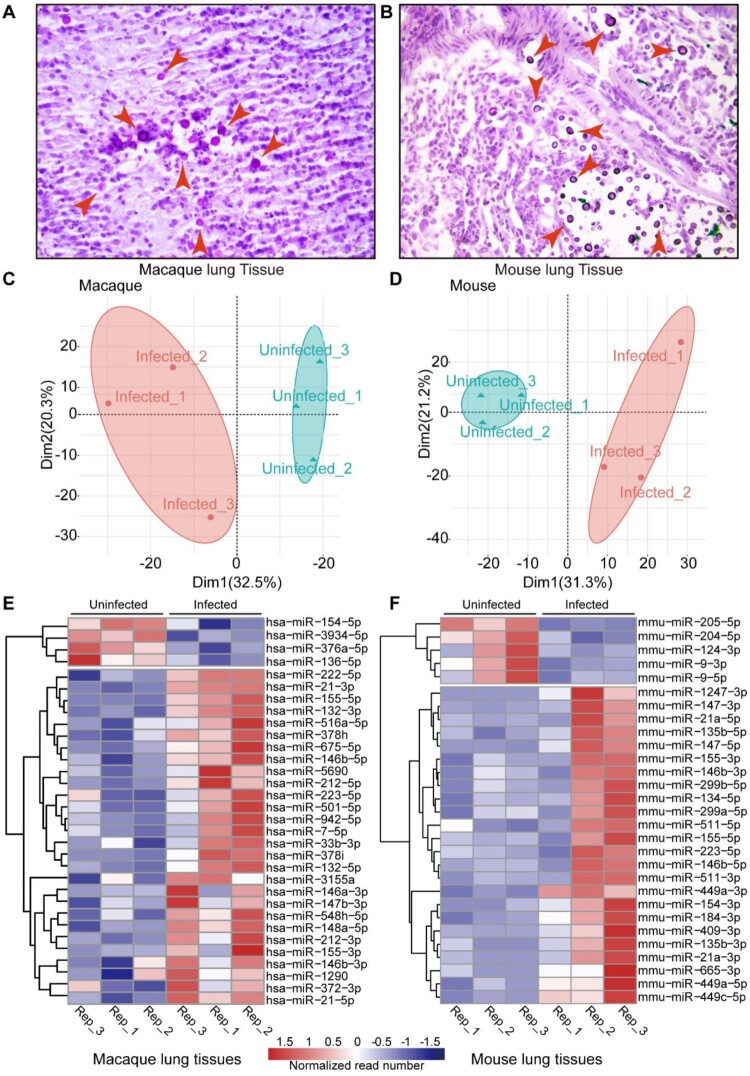
miRNA-Seq of mice and macaques in response to *C. neoformans.* A, B. Histopathology observation of infected macaque and mouse lung tissues. Lung tissues from macaques and mice were fixed, sectioned at 10 µm thickness and stained using mucicarmine. Red arrows indicate *C. neoformans* cells. Scale bar = 10 µm. C, D. PCAs of miRNA-Seq data. Read number of macaques and mice obtained from miRNA-Seq was used for PCA analyses. E F. Heatmaps of differentially expressed miRNAs. miRNAs with *p*-value 0.05 and fold change ≥2 were considered as differentially expressed.

Pathologies of macaque and mouse lung tissues were confirmed by histopathology observations using mucicarmine and haematoxylin/eosin staining to show the capsular structure *of C. neoformans* (Figure 2(A,B)). Total RNAs of macaque and mouse lung tissues were extracted and miRNA transcriptomes were determined. Total and unique reads from omics were calculated, with a number of more than 10^7^ of total read and 10^6^ of unique read (Figure S1A). Reads around 22 nucleotides were the most abundant (Figure S1 B, C). The clean read files were used to map the corresponding genome based on species specificity in miRBase21. However, macaque only has a few miRNA annotations available, therefore, miRNA annotations of humans were employed for macaque samples. As a result, 1038 and 1166 miRNAs were identified in lung tissues from macaques and mice, respectively (Table S1). Principal component analyses (PCA) were examined by using the read number of miRNAs, and both infected and uninfected samples were reproducible (Figure 2C, D). Differentially expressed miRNAs were determined using DESeq. Heatmaps of the differentially expressed items revealed the presence of 32 miRNAs (4 downregulated and 28 upregulated) in macaques and 29 miRNAs (5 downregulated and 24 upregulated) in mice following *C. neoformans* infection, shown as two clusters by column (Figure 2E, F).

### Core responses of the host-derived from miRNA-mRNA integrative analyses

**Figure 3.**
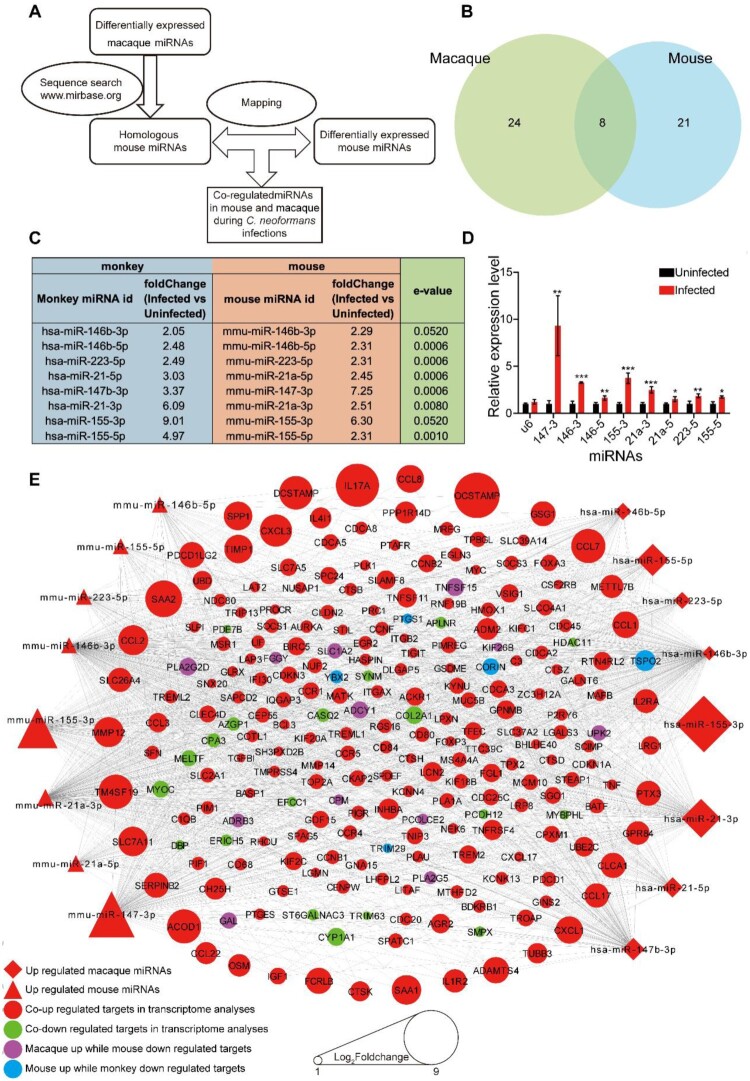
Core regulatory machinery of miRNA-mRNA network during *C. neoformans* infections. A. Schedule of identification of homologous miRNA. miRNAs of macaques were used for searching homologous miRNAs in miRbase. B. Venn diagram of differentially expressed miRNAs. C. Information of eight co-regulated miRNAs. D. RT-qPCR of the eight co-regulated miRNAs in mice. Three biological replicates were performed. An unpaired student’s *t*-test was performed to assess statistical significance; *p < 0.05, ** *p *< 0.01 and ***p < 0.005. E. miRNA-mRNA core regulatory network. Differentially expressed target genes of eight co-regulated miRNAs are shown. The whole network is presented in Figure S3.

To mine and mimic responses in human cryptococcosis, homology analyses were performed (Figure 3A). Eight miRNAs were co-regulated in macaque and mouse during *C. neoformans* infections. Information about the eight miRNAs were displayed, including IDs, foldchanges and homologous e-values, and RT-qPCRs for the eight miRNAs were performed in mice and were consistent with the miRNA-Seq data (Figure 3B, C, D).

**Figure 4.**
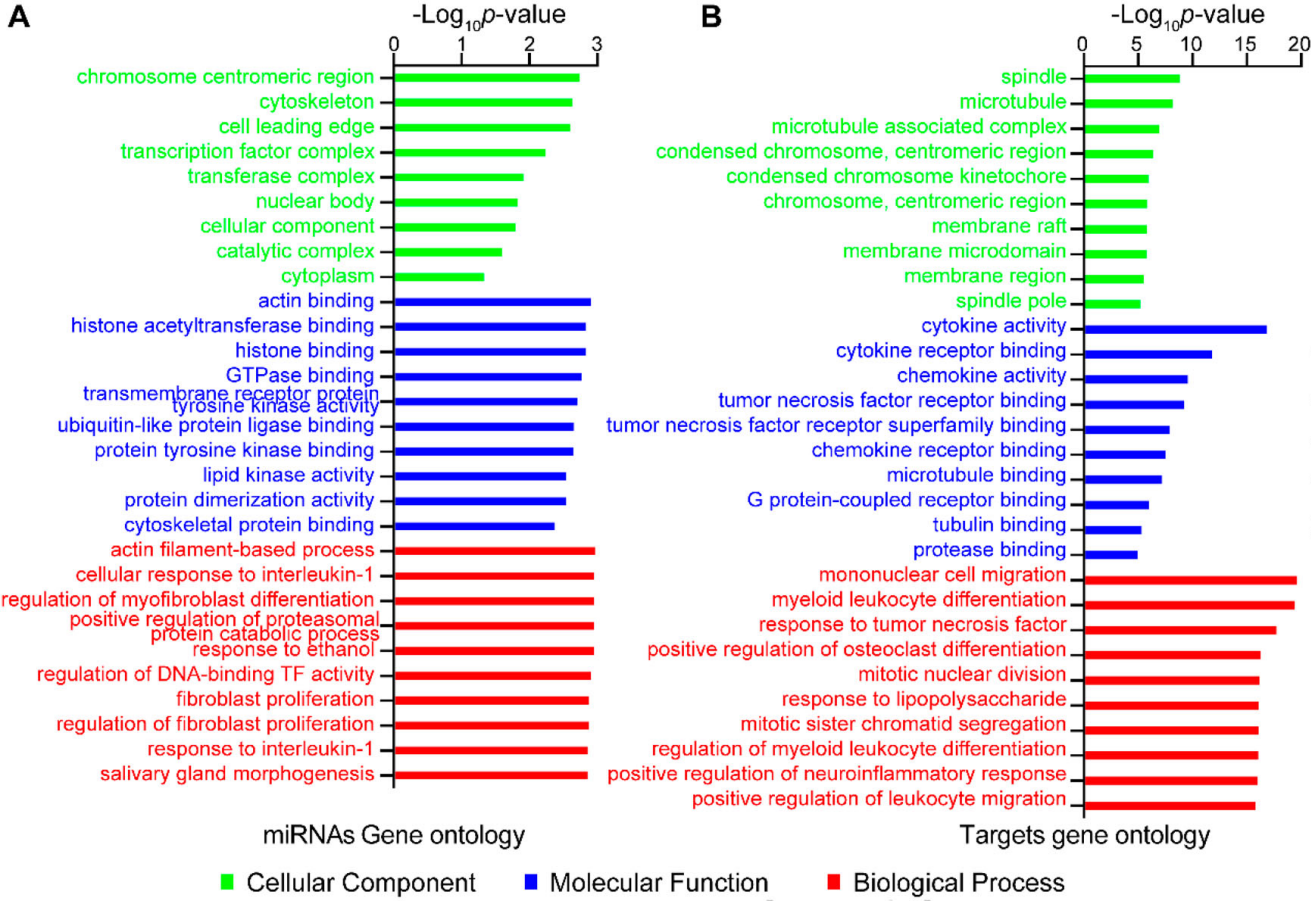
GO analyses of co-regulated miRNAs and mRNAs in macaque and mouse during *C. neoformans* infections. (A) GO terms of miRNAs calculated by miEAA. (B) GO terms of target mRNAs enriched by Clusterprofiler. Eight co-regulated miRNAs and co-regulated mRNAs from Figure 3(E) were subjected to GO analyses, respectively. Top 10 or all significantly enriched GO terms were plotted. Green, blue, and red columns represent cellular component, molecular function, and biological process, respectively.

Targets of the eight co-regulated miRNAs were predicted, then mapped to the RNA-Seq data, and omics of miRNA and mRNA were integrated (Figure S2). The core regulatory target mRNAs, co-regulated by the eight co-regulated miRNAs, were selected for a mini miRNA-mRNA network. This network identified a total of 223 target mRNAs, including OCSTAMP, DC-STAMP, IL17A TNF and LIF, which demonstrated anti-microbial activities (Figure 3E). KEGG and GO analyses were performed on the eight co-regulated miRNAs and targets (Figure 4, Figure S3, Tables S2 and S3). KEGG pathways associated with the immune system, lung diseases, and infectious diseases were significantly enriched at both miRNA and mRNA levels. These pathways included the TNF signalling pathway, Cytokine-cytokine receptor interaction, Osteoclast differentiation, small cell lung cancer, non-small cell lung cancer and Influenza A (Figure S3). GO analyses identified terms involved in cytokine activity, histone, immune cells, monocyte, myeloid leukocyte, and cell cycle were significantly enriched during *C. neoformans* infections (Figure 4). Moreover, actin binding and microtubule and their associated complexes constitute the cytoskeleton system, were highly enriched in GO analyses, including cellular component, molecular function, and biological process. These findings indicated a potential function of the cytoskeleton in cryptococcosis.

### The macrophage “Trojan Horse” was enhanced in patients with HIV/AIDS and could be dampened by a cytoskeleton pathway inhibitor

**Figure 5.**
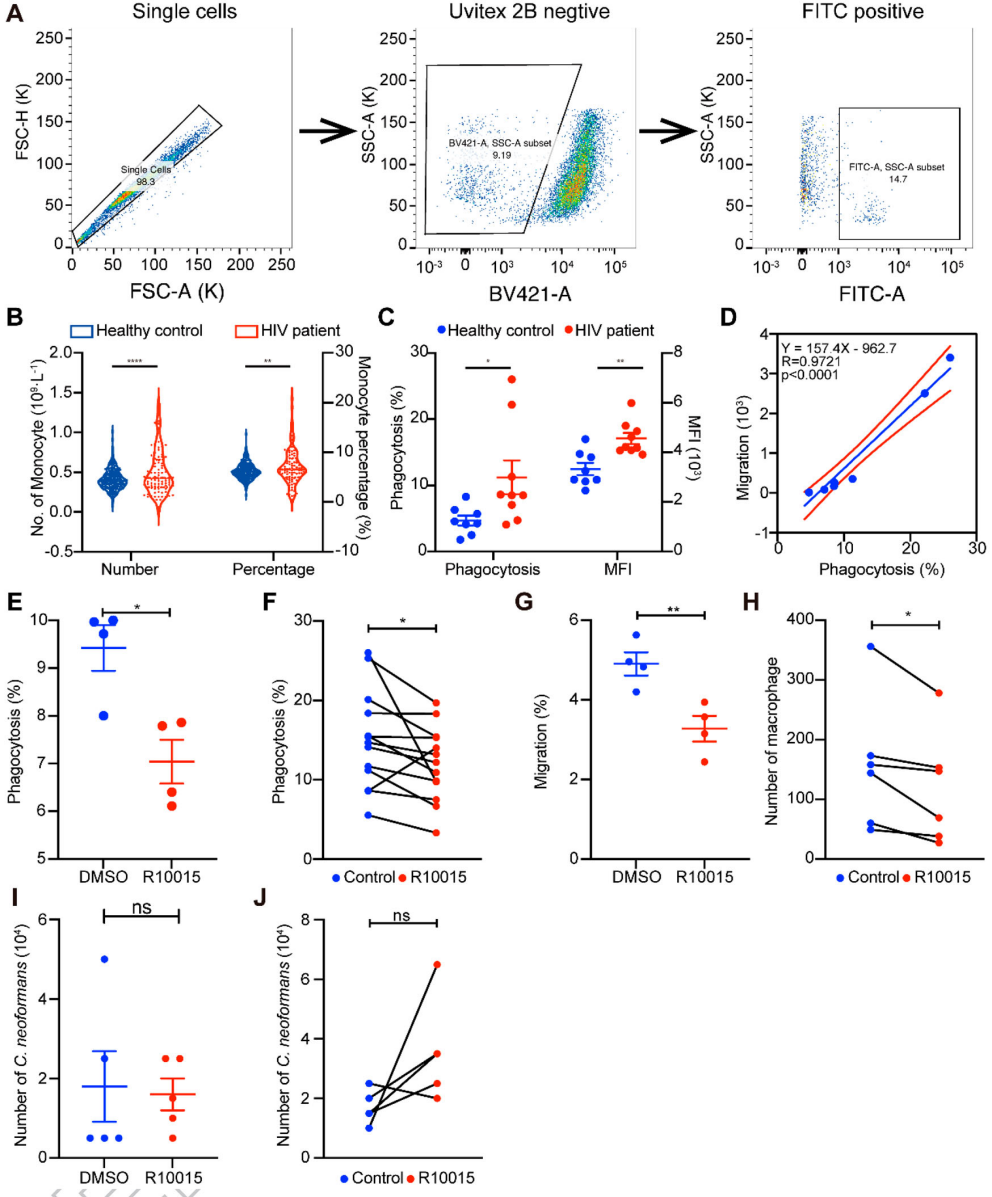
Macrophage “Trojan Horse” was enhanced in patients with HIV/AIDS. (A) Strategies for phagocytosis assessment by flow cytometry. Cryptococcal internalization was determined by flow cytometry using GFP-expressing *C. neoformans* H99. During flow cytometry, single cells were selected and cells with negative Uvitex 2B were considered as phagocytes, of which FITC-positive cells were fungi-internalized cells (Uvitex 2B -/ FITC +). (B) Number and percentage of monocytes between patients with HIV and healthy individuals from clinical data. (C) Phagocytosis effectivity of MDMs from patients with HIV and healthy individuals. (D) Correlation of phagocytosis and migration in MDMs from patients with HIV. E, F. Phagocytosis effectivity was inhibited by R10015 in TDMs (E) and MDMs (F). G, H. Cell migration was dampened by R10015 in TDMs (G) and MDMs (H). I, J. R10015 did not affect killing of *C. neoformans* in TDMs (I) and MDMs (J).

Dysfunctional cytoskeleton of immune cells is one of the major pathological features of patients with HIV/AIDS, who are the predominate population for CM. Based on PSM (Propensity Score Matching, PSM), 100 HIV patients and 200 healthy individuals were compared, which revealed that the number and percentage of monocytes were significantly enlarged in patients with HIV (Figure 5B). To reveal functions of the host cytoskeleton during the battle between host and *C. neoformans*, phagocytosis and transmigration of MDMs from nine patients with HIV/AIDS and eight healthy volunteers were compared (Figure 5A). Phagocytosis effectivity was enhanced in patients with HIV/AIDS (Figure 5C), and furthermore, the lifted MFI (Mean Fluorescent Intensity, MFI) indicated more fungal cells were phagocytosed or underwent more intracellular proliferation in patients with HIV/AIDS. There was no change in transmigration of MDMs between patients with HIV and healthy volunteers (data not shown), but MDM transmigration was positively correlated with phagocytosis capacity (Figure 5D).

To confirm the association of the phenomenon of *C. neoformans* infection in patients with HIV/AIDS with the cytoskeleton, R10015 — a cytoskeleton pathway inhibitor – was employed, who targets LIM Kinase (LIMK) in the cytoskeleton pathway [39]. A growth curve was generated to select the appropriate inhibitory dose of R10015, which was 14.815 µM (Figure S4A). TDMs and human MDMs were pre-treated with R10015 for 2 h, washed and then incubated with opsonized *C. neoformans* overnight. R10015 inhibited the phagocytosis capacity and migration of macrophages derived from both THP-1 cell lines and primary human monocytes (Figure 5E, F and G, H). However, R10015 did not affect the killing in TDMs and MDMs (Figure 5I, J). These results confirmed the vital roles of the cytoskeleton in the macrophage “Trojan Horse.”

### MYOC is an inhibitor for cryptococci brain dissemination by modulating macrophage “Trojan Horse”

**Figure 6.**
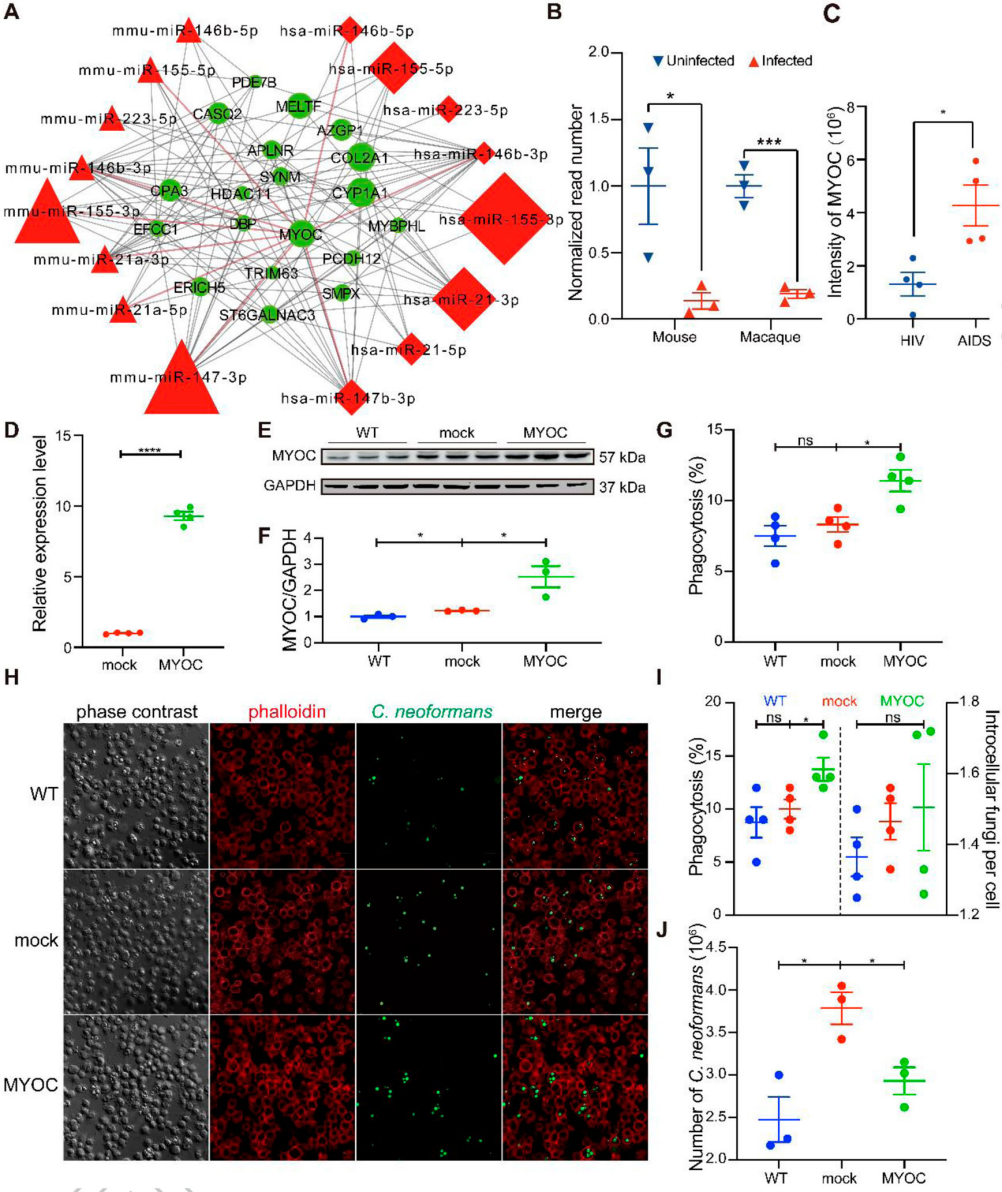
*MYOC* enhanced *C. neoformans* mediated macrophage “Trojan Horse.” A. miRNAs-mRNA network during *C. neoformans* infections. B. Normalized *MYOC* reads in mice and macaques in response to *C. neoformans.* C. Intensity of MYOC protein from comparative proteomes between HIV and AIDS patients. D. Relative expression level of *MYOC* in *MYOC*-gene-edited THP-1 cell lines. Unpaired Student *t* test was performed (n = 4). * *p *< 0.05*.* E. Western blot analysis of MYOC(n = 3). Full blots are shown in Supplementary Figure S4. F. Quantitative analyses of a western blot for MYOC. GAPDH was used as an internal control. Unpaired Student *t*-test was performed (n = 3). * *p *< 0.05*.* G. Effectivity of phagocytosis in *MYOC*-overexpressing TDMs by flow cytometry. Unpaired Student *t* test was performed (n = 4). * *p *< 0.05*.* H. Effectivity of phagocytosis in *MYOC*-edited TDMs by confocal microscopy. TDMs were incubated with GFP-expressed *C. neoformans*, and phagosomes were washed, fixed and stained by F-actin and analyzed through confocal microscopy. Green represents *C. neoformans*, while red represents F-actin. Dots of green in the circle of red indicate phagocyted *C. neoformans* cells. Scale bar = 20 µm. I. Quantitative analyses of phagocytosis capacity by confocal microscopy. TDM cells and fungal cells were counted by imageJ, and the percentage of phagocyted TDMs and the number of intracellular *C. neoformans* were calculated. Unpaired Student *t* test were performed (*n* = 3). * *p *< 0.05*.* J. Killing tests of *MYOC*-overexpressing TDMs*.* Unpaired Student *t* test was performed (*n* = 3). * *p *< 0.05.

To identify functions of the cytoskeleton during *C. neoformans* infections, genes associated with the cytoskeleton were screened, and myocilin was selected. Myocilin is a tubulin-binding protein encoded by the gene *MYOC*, which was one of the centric modulators in miRNA-mRNA regulatory network and was downregulated significantly during *C. neoformans* infections in both macaque and mouse (Figure 6A, B, Table S4). In addition, expression of *MYOC* was elevated in patients with HIV/AIDS (Figure 6C). Previous studies demonstrated that the protein was involved with cell migration and adhesion [40].

To explore functions of *MYOC*, THP-1 cell lines overexpressing *MYOC*-were constructed and expression of *MYOC* was quantified by RT-qPCR and western blotting, which showed a 10-fold and 2-fold induction on mRNA and protein levels compared to mock cells (Figure 6D, E, F, Figure S4B, C). Phagocytosis, migration and killing capacity in *MYOC* gene-edited cell lines were subsequently examined (Figure 6G, H, I, J). Phagocytosis effectivity was increased significantly in TDMs when *MYOC* was overexpressed, either detected by flow cytometry or confocal scanning (Figure 6G, H, I). Killing capacity was also enhanced by *MYOC* overexpression as fewer live *C. neoformans* were detected by CFU assays (Figure 6J). However, no changes were observed in migration assays (Figure S4D). RT-qPCR of genes associated with phagocytosis confirmed the impacts on phagocytes by MYOC, including *CFL1*, *DOCK2*, *RAC1*, *LIMK1,* SCAR/WAVE complex (*BRK1*, *NCKAP1L*, *NHLRC2*), Arp2/3 complex (*ARPC2, ACTR2*) and antifungal effectors (*TLR9*, *ATF3*, *EIF5A*) (Figure S4E). *CFL*, *ATF3*, *EIF5A* and *BRK1* were induced, while *TLR9*, *DOCK2*, *NCKAP1L* and *ARPC2* were repressed (Figure S4E)*.* These indicated that phagocytic and killing genes are affected by the elevated MYOC.

**Figure 7.**
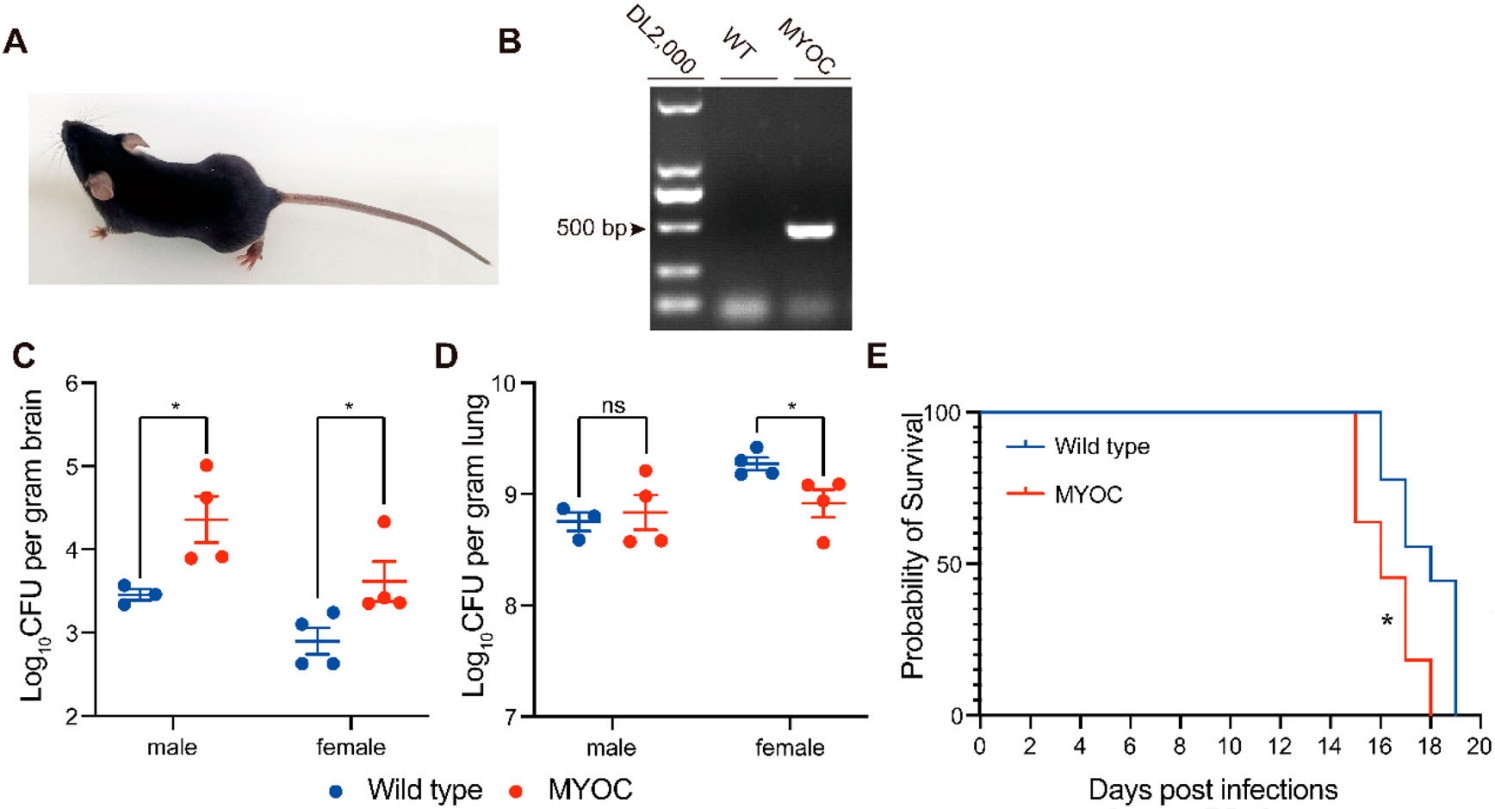
*MYOC* enhanced *C. neoformans* brain invasion in *MYOC*-transgenic mice. A. Photo of *MYOC*-transgenic mice. One of the *MYOC*-transgenic mice at 5 weeks old. B. *MYOC* gene fragment was checked by agarose electrophoresis in *MYOC*-transgenic mice. Genomic DNA was isolated and then PCR and electrophoresis were performed. Target PCR product was 498 bp. C, D. CFU assessments of brain (C) and lung (D) tissues in *MYOC*-transgenic mice. Seven *MYOC*-transgenic mice (three male and four female) and eight wild-type mice (four male and four female) from the same cages were used for CFU assays. An unpaired Student’s *t*-test was performed to assess statistical significance; * *p *< 0.05*.* E. Survival tests of *MYOC*-transgenic mice. Eleven *MYOC*-transgenic mice (five male and six female) and nine wild-type mice (three male and six female) from the same cages were used for survival assays. *Log-rank* (Mantel-Cox) test was employed for statistical analysis; * *p *< 0.05.

To evaluate functions of *MYOC in vivo*, a *MYOC*-transgenic mouse was generated (Figure 7A) and confirmed by PCR (Figure 7B). Six-eight weeks old mice were used for CFU assessments and survival rate determination. Compared to wild-type mice from the same cage, CFUs in brains were enhanced in *MYOC*-transgenic mice in both male and female groups (Figure 7C). CFUs in lungs were decreased in female mice, while no changes in the male group (Figure 7D). Consistent with CFU assays, *MYOC*-transgenic mice showed reduced survival times compared with wildtype mice in survival rate tests (Figure 7E, Figure S4F).

## Discussion

CM is an emerging disease with high mortality, even under current ART conditions, and warrants more attentions in the post-COVID-19 era [1–3,9–12]. Brain dissemination is the lethal infection process. However, clinically relevant mechanisms of brain dissemination in CM are currently limited [13–17]. In this study, the landscapes at transcriptional and post-transcriptional levels were determined in mouse and macaque infection models, which were employed to mimic and unveil responses at miRNA-mRNA regulatory levels in humans. The mouse is the most used animal model for CM in previous studies, but there are marked variations between mice and humans, such as in the process of immune cells maturation. Therefore, data in the current study from *M. fascicularis*, which shows 92.83% sequence identity in humans, was hypothesized be more clinically relevant and may serve as a database for cryptococcosis or mycosis. To investigate core responses during *C. neoformans* infections, GO and KEGG analyses of combined miRNA-mRNA data were performed. Eight key miRNAs were identified, including miR-146a, miR-223 and miR-155, which were induced in monocytes by co-cultured with *C. neoformans in vitro* [32]. The functions of unique miRNAs were not explored in the current study, instead, the cytoskeleton pathway was characterized as a core regulatory modulator based on enrichment analyses of miRNAs and their targets.

Our data identified the fundamental roles of the cytoskeleton during macrophage “Trojan Horse” formation, and furthermore highlighted potential targets for novel “orphan drug” development. Studies have indicated relationships between cytoskeleton and fungal infections [23,41–44]. However, the mechanisms of interaction have yet to be elucidated and there is a lack of *in vivo* studies confirming the relationship. Important roles of “Trojan horse” by macrophages during fungal CNS invasion have previously been reported, whose processes were cytoskeleton-dependent [45]. The current study illustrated the basic functions of cytoskeleton dynamics on capacities of phagocytosis and migration of macrophages, which can be blocked by cytoskeleton inhibitors. However, small-molecule drugs, such as vanadate, cytochalasin D, Y27632 and R10015 are often toxic to humans [23,39,46]. This toxicity involves fundamental roles of innate immunity and acquired immunity of macrophages, NK cells and T cells, which also play important roles during fungal, bacterial and viral infections. Overcoming toxicity of cytoskeletal inhibitors is the main barrier to clinical application.

Further analyses revealed that a dysfunctional cytoskeleton may account for the high prevalence of CM in patients with HIV/AIDS. Studies indicated a similar variation of cytoskeleton pathway during HIV infection and *C. neoformans* or *A. fumigates* infections [23,42,43,47,48]. Indeed, *C. neoformans* is an environmental yeast that is globally ubiquitous and easily accessible for all individuals [49]. However, why do these happen much more in HIV/AIDS? Previous studies suggested the decreased number of CD4 T cells was a high-risk factor; however, this is contradictory to the high prevalence of high-level CD4 T cells who are ART experienced [50–52]. Another hypothesis is the increased exposure to a *C. neoformans* environment, but this is inconsistent with the low prevalence observed in other people in the same environment [53]. Data from the current study suggest the dampened cytoskeleton structure may be the reason for the high prevalence of CM in patients with HIV. In patients with HIV/AIDS, numerous studies have proved the cytoskeleton was dampened in immune cells of PBMCs [39,48,54], which contributes to fungal invasion of the CNS by the macrophage “Trojan Horse.” Furthermore, vomocytosis was recently found to be enhanced by HIV infection in macrophages [55], which process was also regulated by the cytoskeleton.

In addition, the current study identified *MYOC* as a novel modulator for a fungal invasion via regulation on macrophages. Our data showed repression of MYOC in *C. neoformans* infections and induction in HIV infection. Myocilin co-localizes with microtubules, the endoplasmic reticulum (ER), and the Golgi apparatus [56,57], and overexpression of *MYOC* induces a loss of actin stress fibres [58]. Furthermore, myocilin promotes cell migration and phagocytic activities of human trabecular meshwork cells [40,59,60]. In the present work, *MYOC* overexpressed THP-1 cell line was generated, and expression of genes associated with actin polymerization and phagocytosis indicated an enhanced phagocytosis and killing capacity in *MYOC*-overexpressed THP-1 cells. Moreover, overexpression of *MYOC* in TDMs elevated phagocytic activities and migration, which induced more intracellular cryptococci and enhanced effects of the macrophage “Trojan Horse.” This indicated that *MYOC* may be the effector for cryptococcoses secondary to HIV/AIDS.

**Figure 8.**
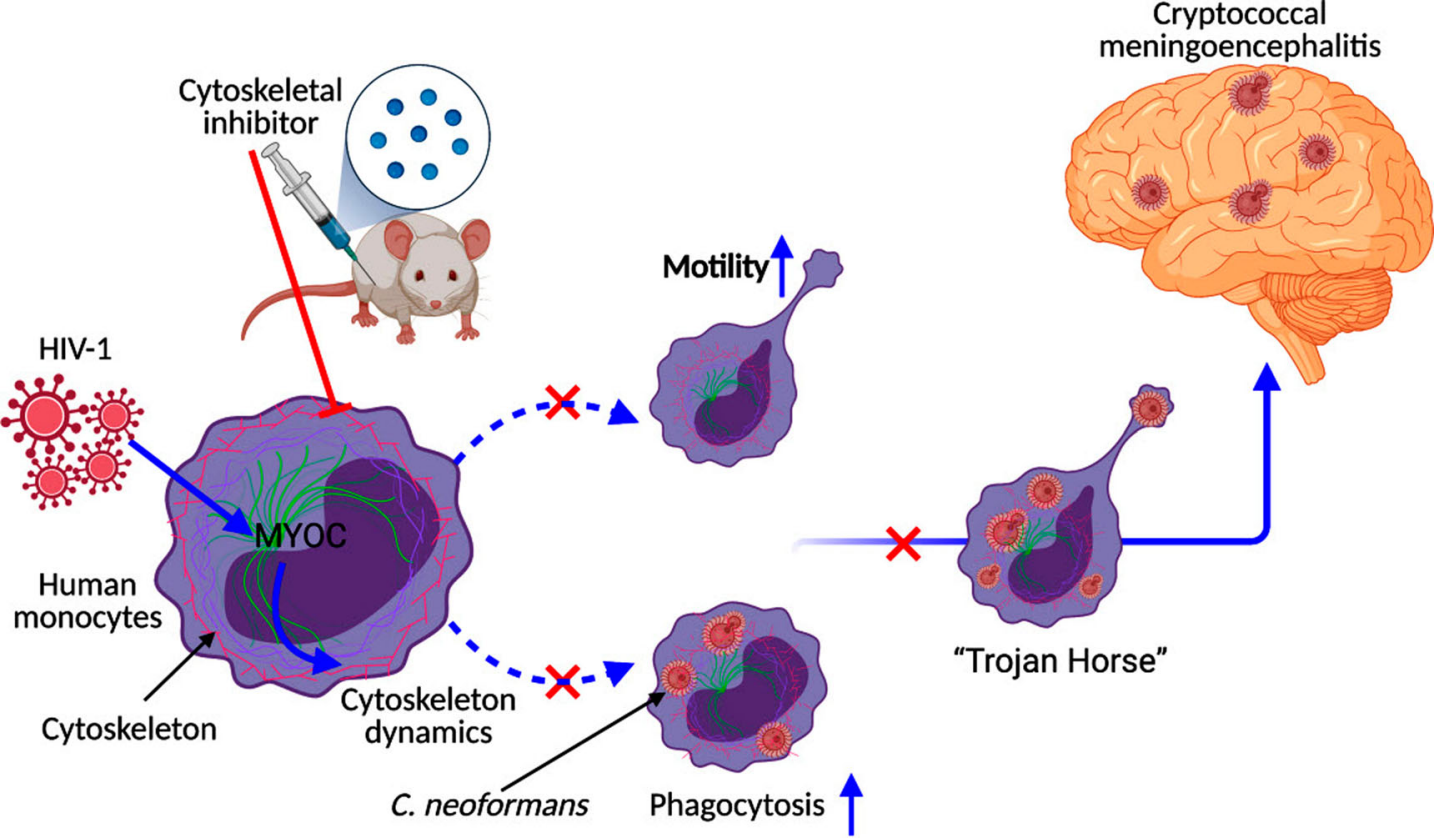
Conceptual graph of HIV-triggered CM. HIV-1 infections trigger cytoskeleton dynamics by induction of MYOC protein, enhance the macrophage “Trojan Horse” and induce CM (shown in blue lines). Inhibition of cytoskeleton pathways or *MYOC* dampens the “Trojan Horse” and decreases the number of cryptococci in the brain (shown in red lines). Figure 7 was created with BioRender.com.

In conclusion, this study described global miRNA-mRNA regulatory responses during *C. neoformans* infections in primate and rodent animal models, which serves as a clinically relevant database for fundamental and clinical research. We highlight the importance of *MYOC* and cytoskeleton pathways during *Cryptococcus* meningoencephalitis and underscores their critical functions in the formation of “Trojan Horse” (Figure 8). This study demonstrates the critical roles of the cytoskeleton in fungal CNS invasion, reveals a potential direct explanation for the high prevalence of CM in patients with HIV/AIDS, and may facilitate the development of novel anti-fungal drugs.

## Supplementary Material

Supplemental Material

## Data Availability

The RNA-Seq raw data files were previously deposited in the National Center for Biotechnology Information (NCBI) Gene Expression Omnibus (GEO) with GEO Series accession ID GSE122785 [22]. Raw data of microRNA-Seq is available to researchers and can be provided upon request. This manuscript has been posted to a preprint server bioRxiv with the DOI: https://doi.org/10.1101/2022.02.14.480.319.
