# Supplementary figures and images for: Comparative miRNA transcriptomics of macaques and mice reveals MYOC is an inhibitor for Cryptococcus neoformans invasion into the brain
Source: Emerg Microbes Infect. 2022 Jun 4;11(1):1572–85. doi: 10.1080/22221751.2022.2081619 (PMC9176638; doi:10.1080/22221751.2022.2081619)

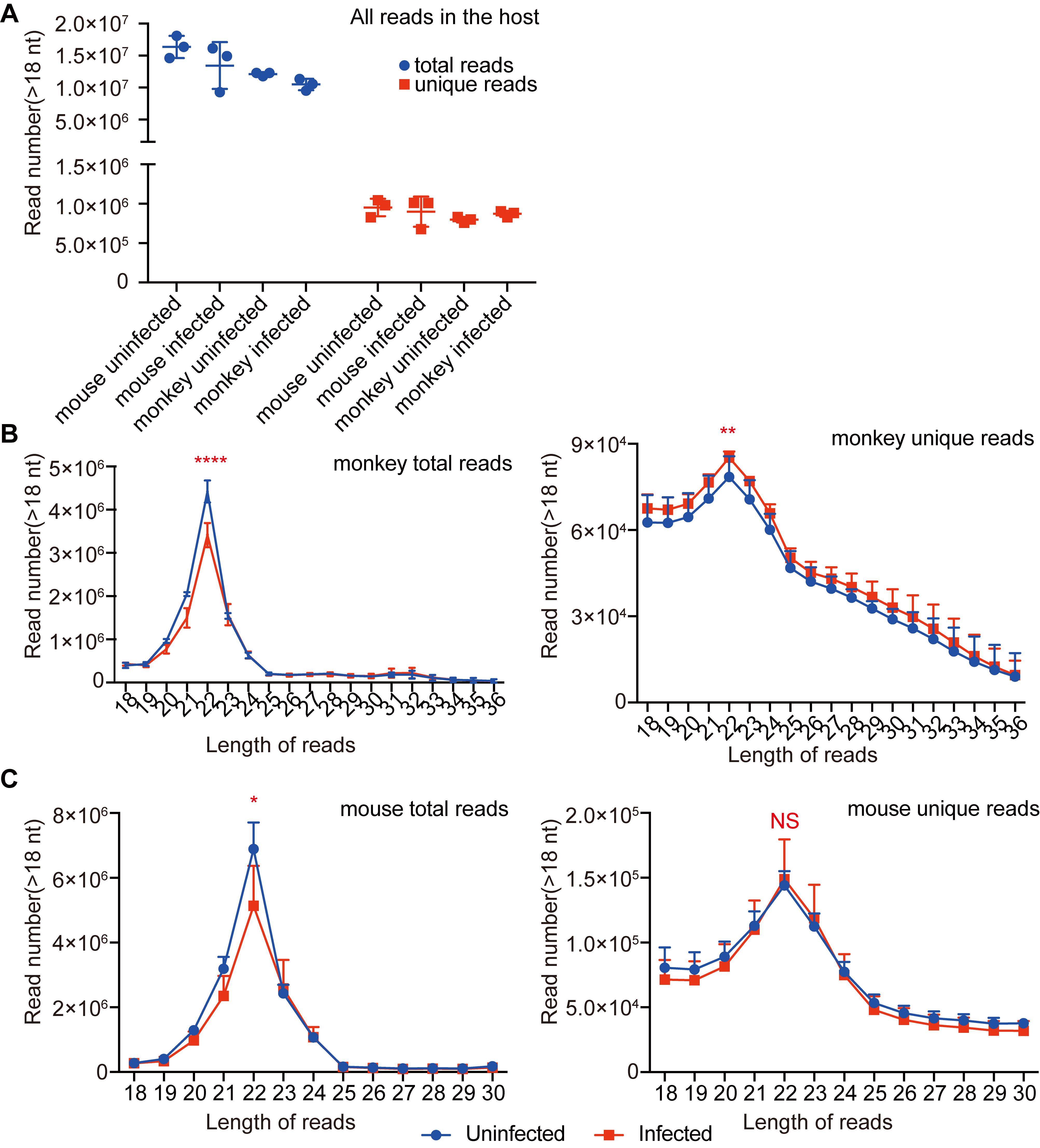

Supplement: Supplemental Material [file TEMI_A_2081619_SM5657.zip › Figure S1.tif]

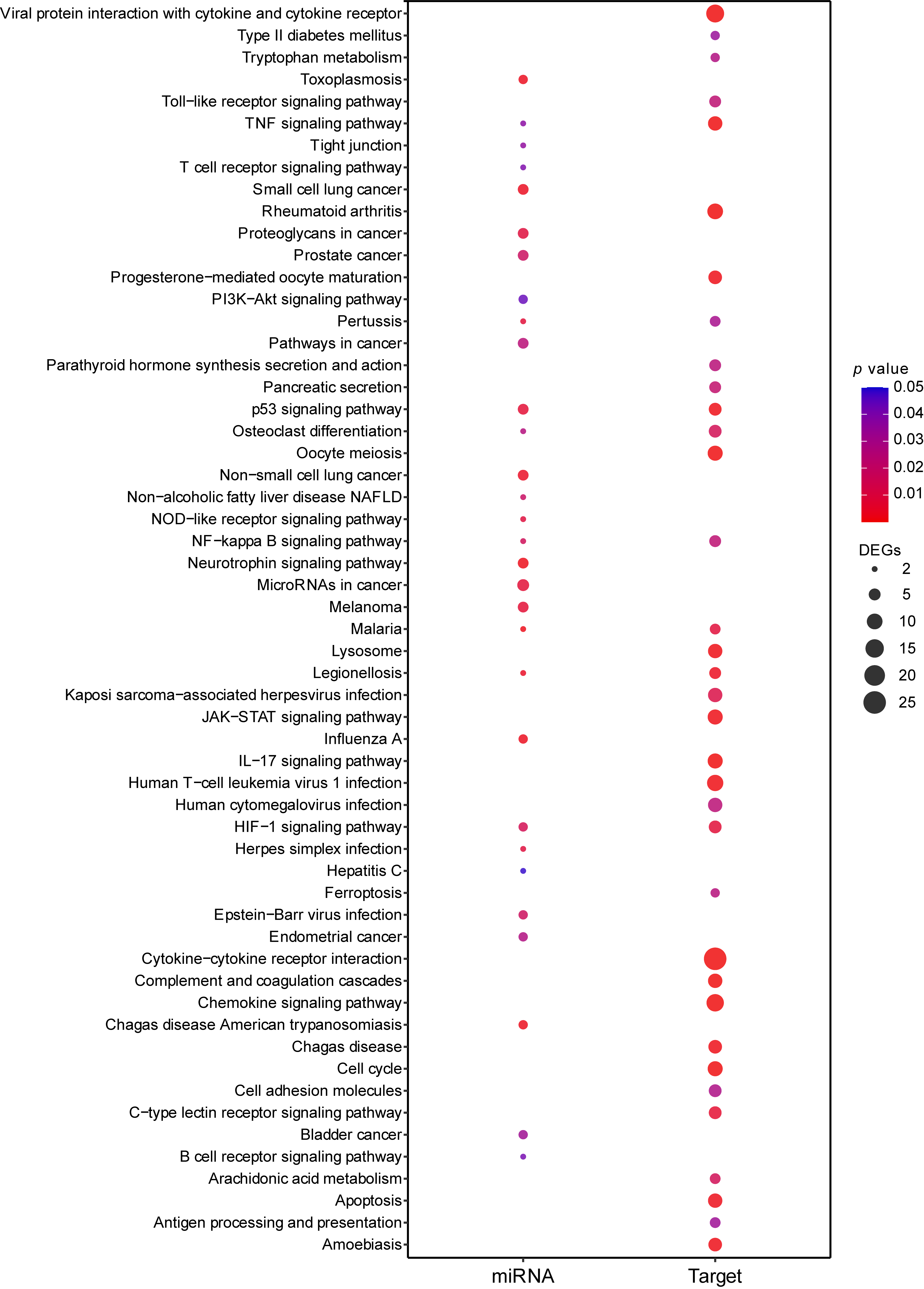

Supplement: Supplemental Material [file TEMI_A_2081619_SM5657.zip › Figure S3 KEGG.tif]

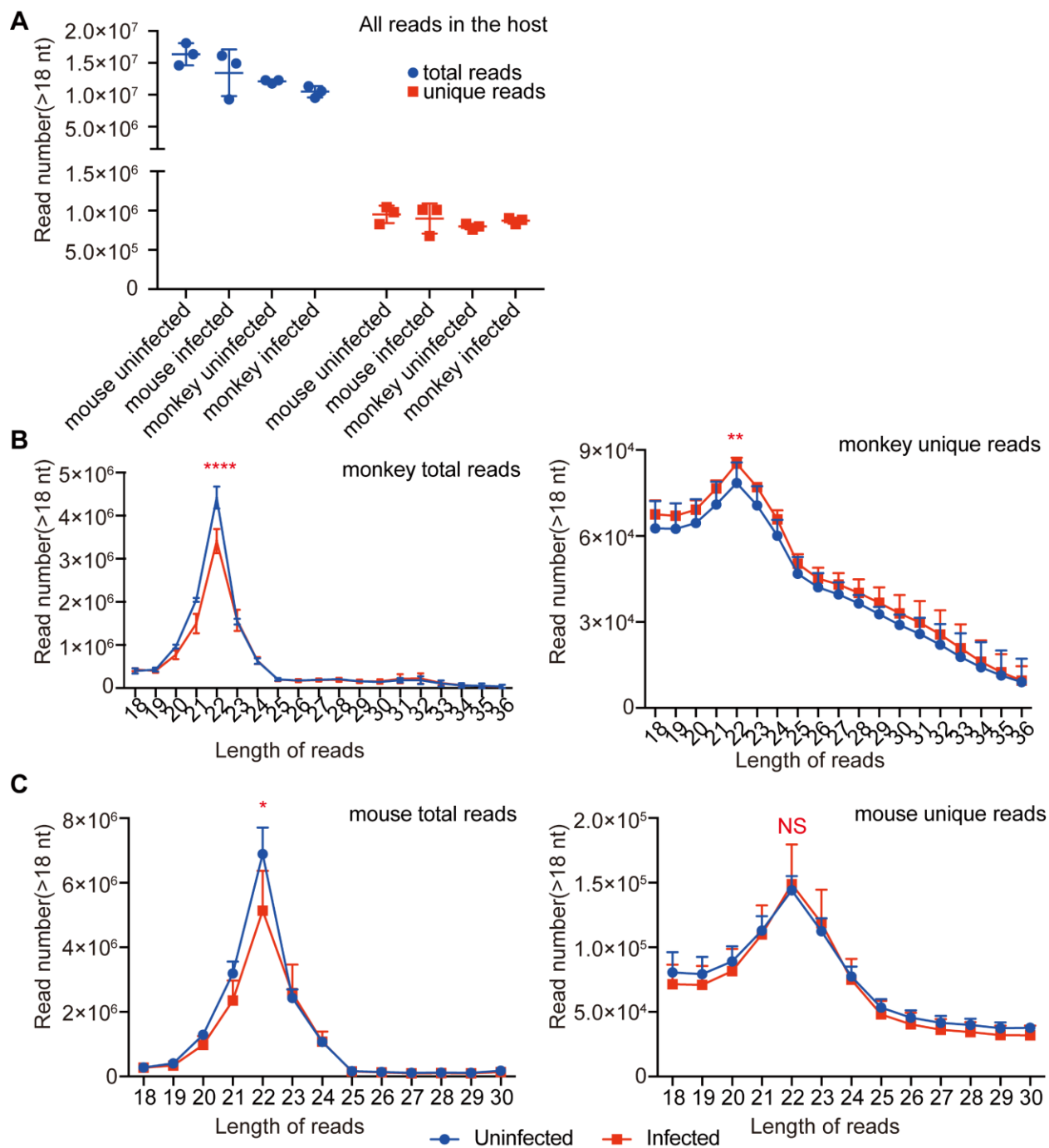

Figure S1

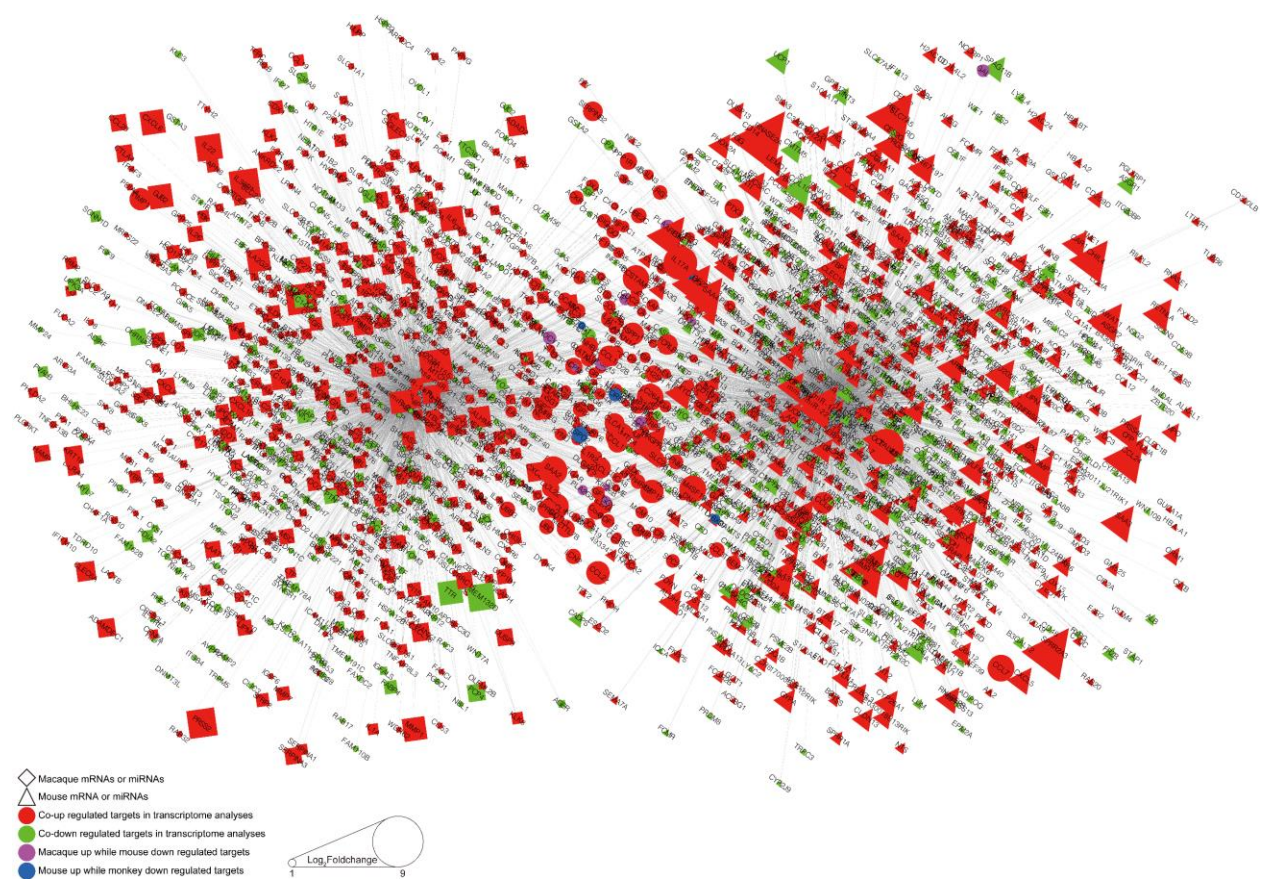

Figure S2

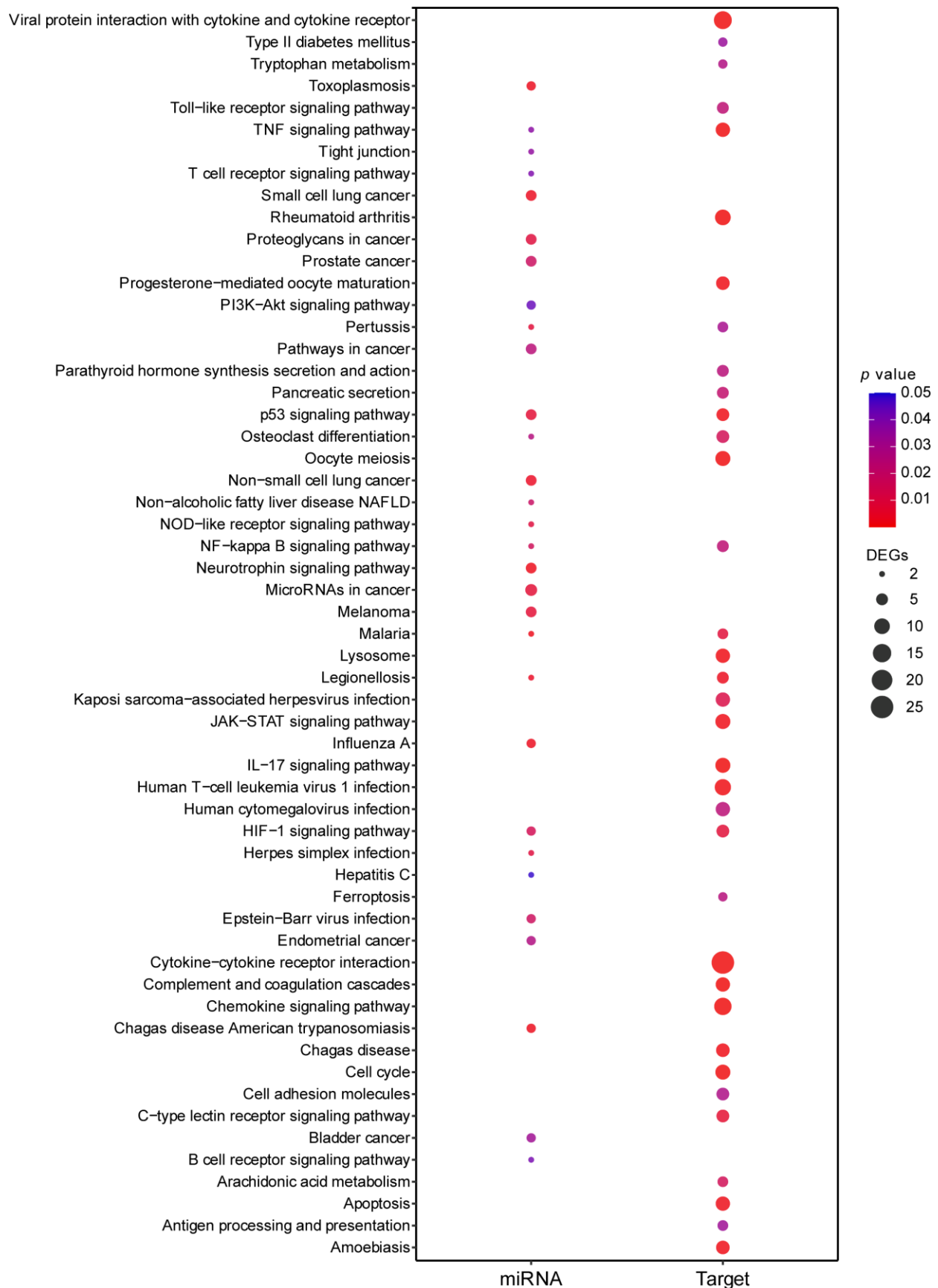

Figure S3

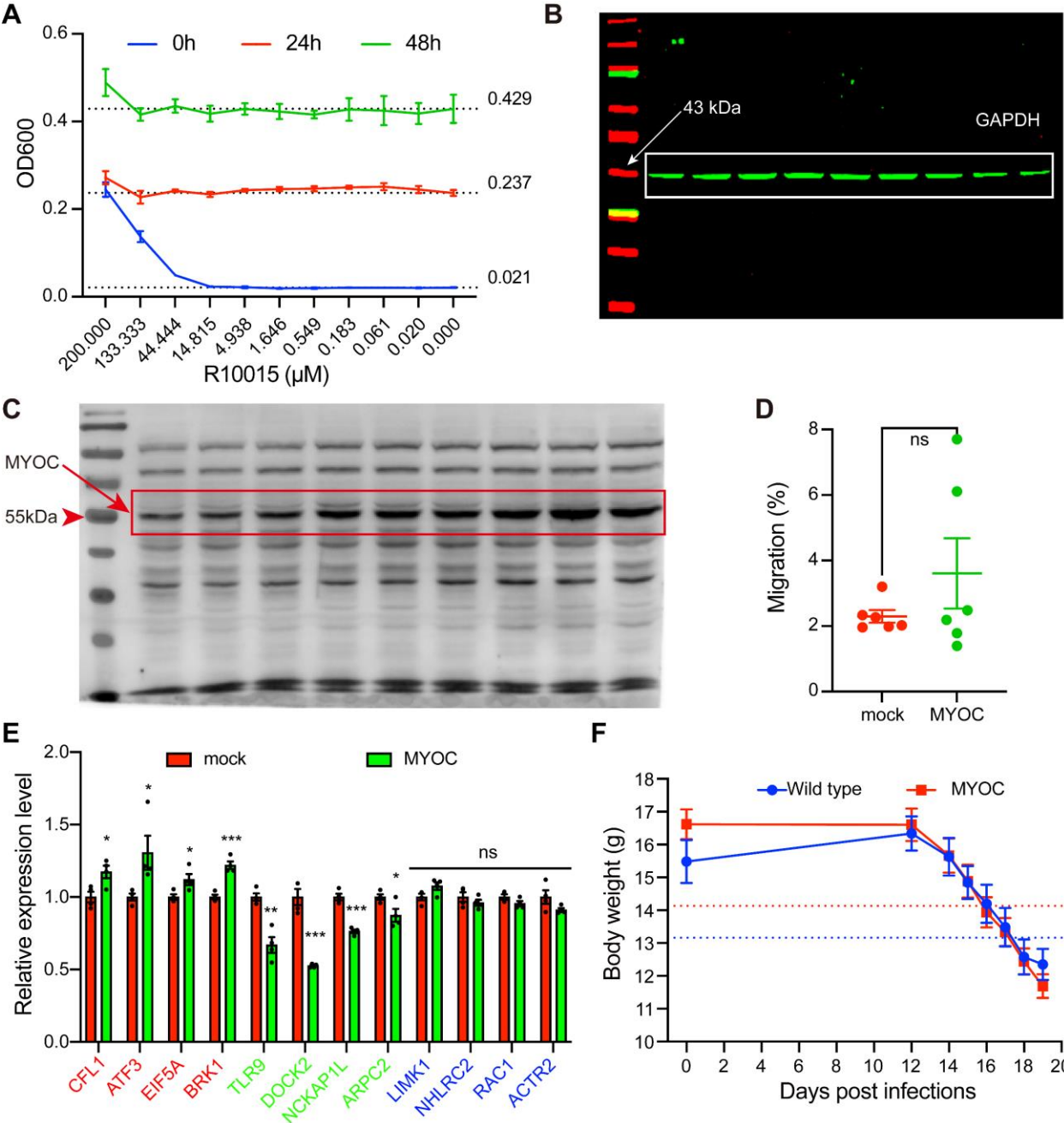

Figure S4

Supplement: Supplemental Material [file TEMI_A_2081619_SM5657.zip › Supplementary Figures.pdf]
